# Phytochemical Profile and In Vivo Assessment of Toxicity and Anti-Inflammatory Activity of *Cenostigma pluviosum* var. *peltophoroides* (Benth.) Gagnon & G.P. Lewis

**DOI:** 10.3390/plants15101508

**Published:** 2026-05-15

**Authors:** Natanael Teles Ramos de Lima, Gabriela Ribeiro de Sousa, Gustavo Gomes da Silva, Geovana Ferreira Guedes Silvestre, Alan Ferreira Alves, Ivana Maria Fechine, Maria de Fatima Agra, Alisson Macário de Oliveira, Josean Fechine Tavares, Marcelo Sobral da Silva, José Maria Barbosa Filho

**Affiliations:** 1 Postgraduate Program in Natural and Synthetic Bioactive Products, Federal University of Paraíba, João Pessoa 58051-900, Brazil; nteles@ltf.ufpb.br (N.T.R.d.L.); geovana.silvestre@ltf.ufpb.br (G.F.G.S.); alvesalanx@gmail.com (A.F.A.); josean@ltf.ufpb.br (J.F.T.); marcelosobral.ufpb@gmail.com (M.S.d.S.); 2 Postgraduate Program in Pharmaceutical Sciences, State University of Paraíba, Campina Grande 58429-500, Brazil; grsousafarm@gmail.com; 3 Department of Pharmacy, Federal University of Rio Grande do Norte, Natal 59078-970, Brazil; gustavogmaltas@gmail.com (G.G.d.S.); alisson.macario@ufpe.br (A.M.d.O.); 4 Department of Pharmacy, State University of Paraíba, Campina Grande 58429-500, Brazil; ivana.fechine@servidor.uepb.edu.br; 5 Program of Biotechnology, Center for Biotechnology, Federal University of Paraíba, João Pessoa 58051-900, Brazil; agramf@ltf.ufpb.br; 6 Department of Pharmaceutical Sciences, Health Sciences Centre, Federal University of Paraíba, João Pessoa 58051-900, Brazil

**Keywords:** Fabaceae, sibipiruna, tannins, HPLC-ESI-MS/MS, metabolomics, phytocomplex, mutagenic effect, inflammation, ethnopharmacology

## Abstract

*Cenostigma pluviosum* var. *peltophoroides*, known as “sibipiruna,” is a plant rich in polyphenols used in traditional medicine for gastrointestinal disorders. The study aimed to investigate the chemical composition of the crude ethanolic extract of the stem bark (CEECP), evaluating its in vivo toxicity, genotoxicity, mutagenicity and anti-inflammatory activity. The plant material was macerated in 95% ethanol for 72 h, and the solvent was removed by rotary evaporation to obtain CEECP. Chemical characterization was performed by HPLC-ESI-MS/MS in negative mode. In vivo approaches were performed using male/female Swiss albino mice. Acute toxicity was assessed at a single high dose of 2000 mg/kg. Mutagenicity was investigated by the micronucleus test and genotoxicity by the comet assay, both at a dose of 2000 mg/kg. Anti-inflammatory activity was evaluated in carrageenan-induced paw edema and peritonitis models, at doses of 50, 100, and 200 mg/kg. HPLC-ESI-MS/MS analysis showed the presence of hydrolyzable tannins, phenolic acid heterosides, and biflavonoids. The safety profile of the CEECP was demonstrated for the first time, with no evidence of acute toxicity, mortality, mutagenicity, or genotoxicity at the tested doses. The extract significantly reduced paw edema in a dose-dependent manner at doses of 100 and 200 mg/kg, with inhibition rates of 65.78% and 73.12%, respectively, and also decreased leukocyte migration in the peritonitis model by 61.81% and 72.79% at the same doses. These findings indicate the CEECP as a source of pharmacologically relevant phytocompounds and, most notably, demonstrate its pronounced anti-inflammatory activity. Furthermore, the extract exhibited a favorable safety profile in the toxicological evaluations, highlighting the extract as a promising anti-inflammatory agent.

## 1. Introduction

Inflammatory diseases represent a major global health problem, encompassing a wide range of illnesses and infectious processes. One of their main characteristics is the presence of pain, which significantly affects the quality of life of affected individuals [1]. Although conventional pharmacological treatments are widely used to manage inflammation and pain, their prolonged use is often associated with significant adverse effects, limiting their long-term application [2]. In this context, the continuous search for new bioactive compounds with anti-inflammatory and analgesic potential has become increasingly important, aiming to offer safer and more effective alternatives for improving health.

Medicinal plants are a fundamental resource for human health, especially in regions where access to drugs remains limited. In recent decades, there has been a growing scientific and social interest in identifying safer therapeutic alternatives derived from natural sources [3,4]. However, despite their widespread use, plant-derived products are not necessarily free of toxic effects, and their complex chemical composition can significantly influence their biological activity and safety profiles [5]. Therefore, rigorous toxicological evaluation is essential to establish safe dosage ranges and identify potential adverse effects, including organ toxicity, and genotoxic or mutagenic damage [6].

In this context, the genus *Cenostigma* has attracted increasing attention due to its ethnomedicinal relevance and phytochemical diversity. Historically included in Caesalpinia sensu lato (Fabaceae, subfamily Caesalpinioideae), recent phylogenetic advances have led to its taxonomic reclassification, although a specific portion of the available biological data remains associated with its former designation. Species belonging to this group have been traditionally used in the treatment of various conditions, including malaria, fever, respiratory infections, dysentery, inflammatory disorders, and parasitic diseases [7,8].

*Cenostigma pluviosum* var. *peltophoroides* (Fabaceae), a Brazilian endemic species popularly known as “sibipiruna,” occurs predominantly in the Atlantic Forest and Pantanal biomes and is widely used for ornamental purposes [9,10]. In folk medicine, the stem bark has been used to treat gastrointestinal disorders such as diarrhea and dysentery [11]. Previous studies report various biological activities for this species, including antimicrobial, antimalarial, antifungal, antiviral, and wound-healing effects, highlighting its pharmacological potential [12,13,14,15,16].

Despite these reports, there is a notable lack of comprehensive studies addressing both the pharmacological efficacy and the safety profile of this species. To our knowledge, this is the first study to simultaneously investigate the anti-inflammatory activity, the in vivo toxicological profile, and the genotoxic and mutagenic potential of the ethanolic extract obtained from the stem bark of *C*. *pluviosum*. Furthermore, this work provides a detailed phytochemical characterization of this species by HPLC-ESI-MS/MS, expanding current knowledge about its chemical composition.

In this context, the present study aims to evaluate its anti-inflammatory effects, assess its safety through behavioral, hematological, and biochemical parameters, and investigate potential genotoxic risks, thus contributing to the scientific validation of its traditional use and supporting the development of safer phytotherapeutic agents.

## 2. Results

### 2.1. Phytochemical Characterization by HPLC-ESI-MS/MS

**Figure 1:**
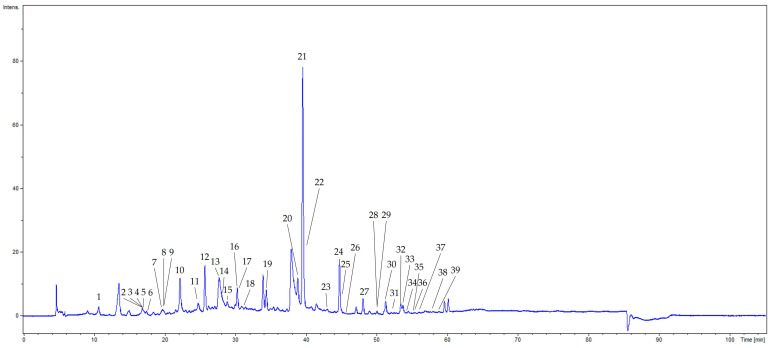
Chromatogram at 254 nm of crude ethanolic extract of the stem bark of *Cenostigma pluviosum* by HPLC–DAD–ESI–MS^n^.

A total of 39 compounds were identified by HPLC-ESI-MS/MS. The total ion chromatogram of the stem bark extract of *Cenostigma pluviosum* is shown in Figure 1. Data concerning the identification of the peaks are summarized in Table 1, in which we report the retention time, the *m*/*z* value of the deprotonated ion, molecular formula and the calculated *m*/*z* value, mass error expressed in ppm, fragmentation data provided and the proposed chemical identity (annotation).

LC-MS analysis of CEECP revealed a chemical profile characterized by a high abundance of phenolic compounds. Representative molecules included hydrolyzable tannins, phenolic acids, lignans, and flavonoids, with a notable presence of biflavonoids. Ellagitannins and gallotannins were the predominant classes, including complex structures containing galloyl and hexahydroxydiphenoyl (HHDP) groups. Additionally, several simple phenolic acids were identified, such as gentisic, vanillic, and syringic acids, mainly in glycosylated forms. The profile also included derivatives of ellagic acid, many of which were methylated.

### 2.2. Acute Oral Toxicity

**Figure 2:**
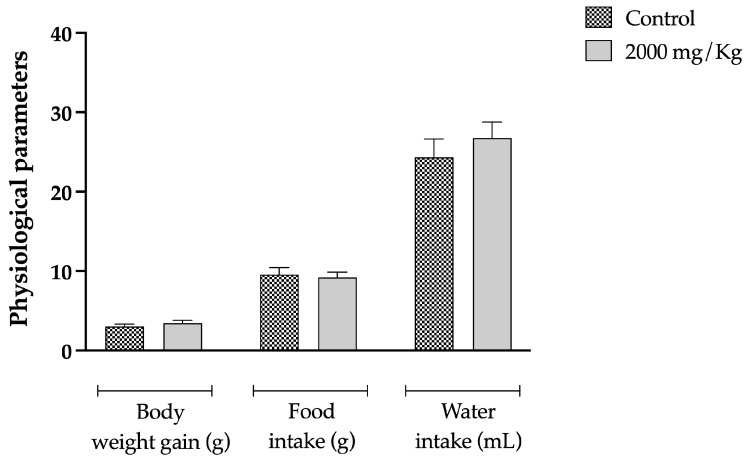
Body weight gain, food and water consumption of mice orally treated with CEECP during the toxicity study. Values expressed as mean ± SD (*n* = 5/group). No significant differences (*p* > 0.05) were found in comparison with the control.

Toxicological evaluation of CEECP demonstrated no significant alterations (*p* > 0.05) in the general, hematological, biochemical, and morphological parameters evaluated in the acute toxicity protocol (2000 mg/kg). Taken together, the values indicate that, under the experimental conditions employed, CEECP did not promote detectable signs of systemic toxicity (Figure 2 and Table 2 and Table 3). No differences were observed in body weight gain, feed consumption or water intake, in hematological parameters, including erythrocyte and leukocyte counts, hematimetric indices and leukocyte differential, or biochemical markers of renal function, liver function, protein profile, and total cholesterol.

In addition, analysis of the relative weight of vital organs, such as the liver, kidneys, lungs, heart, and spleen, did not show any treatment-related changes at doses of 2000 mg/kg (Table 4).

### 2.3. Genotoxicity

In the micronucleus test evaluation (Table 5), treatment with CEECP at a dose of 2000 mg/kg did not significantly increase the number of micronucleated polychromatic erythrocytes (MNPCE), and the PCE/NCE ratio in the treated group remained comparable to the negative control (*p* > 0.05).

The positive control (DXR) significantly increased MNPCE frequency (28.2 ± 2.4), whereas CEECP did not alter the PCE/NCE ratio compared to the untreated control, indicating the absence of myelotoxicity and mutagenicity, and no induction of chromosomal or bone marrow damage in mice.

Similarly, in the comet assay (Table 6), CEECP did not increase the total frequency of damaged cells nor alter the distribution of damage classes relative to the negative control (*p* > 0.05). The average damage score also remained similar to the control. Class 0 cells were predominant in the treatment with CEECP in peripheral blood collected after 24 h, while the group treated with doxorubicin showed a significant increase in the number of damaged cells, a higher proportion of damage classes 2 and 3, and a significant increase in the total score (*p* < 0.05) (79.22), which validates the experimental data of this study.

### 2.4. Anti-Inflammatory

**Figure 3:**
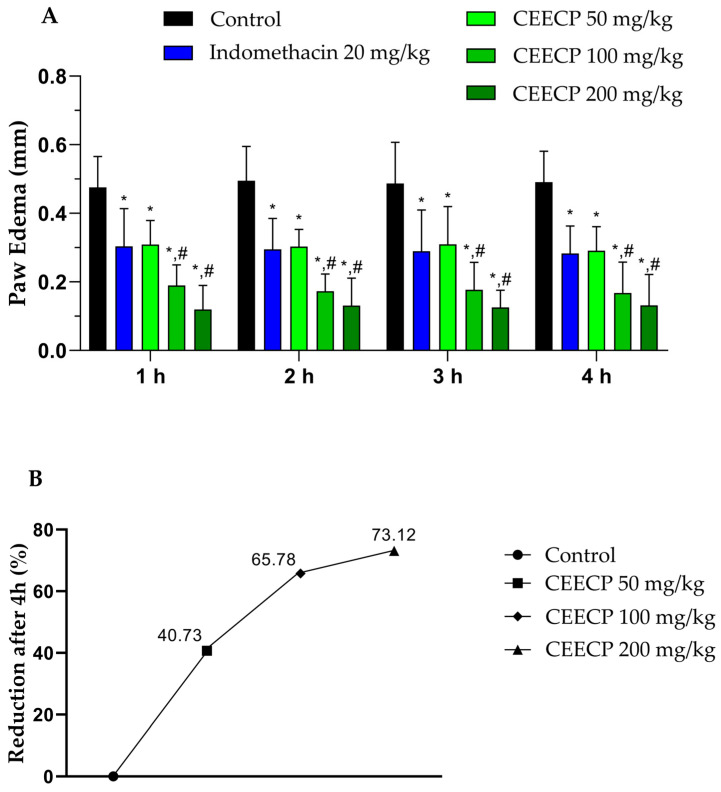
Effect of CEECP on carrageenan-induced hind paw edema in mice. (**A**) Percentage inhibition of paw edema over time and (**B**) percentage reduction after 4 h. Data are expressed as mean ± SEM (*n* = 6). * Significantly different from the control (vehicle) and # significantly different from the indomethacin-treated group (ANOVA followed by Bonferroni post hoc test, *p* < 0.05).

The anti-inflammatory effect of CEECP is shown in Figure 3. The sustained pronounced and progressive edema over the 4 h in the negative control group (untreated) confirms the adequate induction of the inflammatory response and validates the experimental model. Complementarily, the positive control group treated with Indomethacin (20 mg/kg) showed significant and consistent inhibition of edema (*p* < 0.05), demonstrating the sensitivity of the protocol in detecting compounds with anti-inflammatory activity.

Administration of CEECP significantly reduced carrageenan-induced paw edema in a dose-dependent manner. As shown in Figure 3B, CEECP produced inhibition of 40.7%, 65.8%, and 73.1% at doses of 50, 100, and 200 mg/kg, respectively, after 4 h, compared with the control group. Indomethacin showed 42.36% inhibition, which was statistically significant only relative to the vehicle group. Notably, edema was significantly reduced (*p* < 0.05) in all CEECP-treated groups from the first hour after injection, indicating an early onset of anti-inflammatory activity.

**Figure 4:**
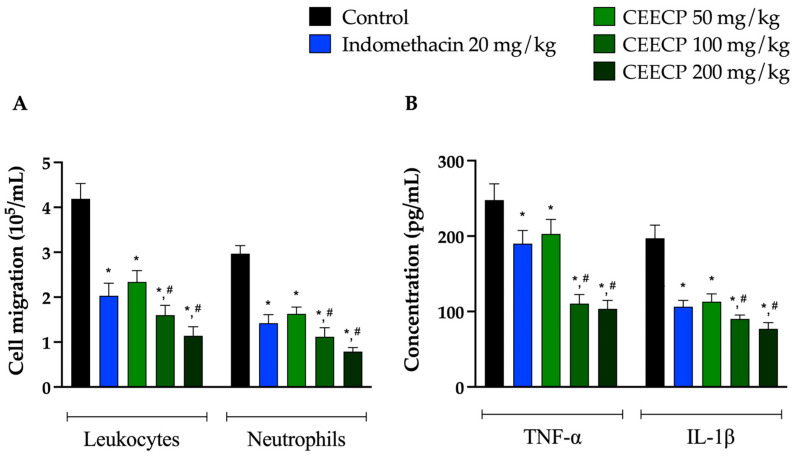
Effect of *C. pluviosum* on leukocyte and neutrophil migration (**A**) in carrageenan-induced peritoneal exudate and on TNF-α and IL-1β levels (**B**) in mice. Data are expressed as mean ± SEM (*n* = 6). * Significantly different from the control (vehicle) and # significantly different from the indomethacin-treated group (ANOVA followed by Bonferroni post hoc test, *p* < 0.05).

Similarly, CEECP significantly reduced total leukocyte and neutrophil migration in the carrageenan-induced peritoneal exudate model in a dose-dependent manner. At 100 mg/kg, the extract inhibited leukocyte and neutrophil migration by 61.81% and 62.29%, respectively, while at 200 mg/kg, the inhibition reached 72.79% for leukocytes and 73.40% for neutrophils. Notably, these effects were greater than those observed with indomethacin. Furthermore, CEECP significantly decreased the levels of tumor necrosis factor-α (TNF-α) and interleukin-1β (IL-1β) (Figure 4).

In general, the 50 mg/kg dose showed moderate anti-inflammatory activity in both experimental models, promoting significant reductions when compared to the control group. In the paw edema assay, even at the lowest dose tested, CEECP demonstrated a relevant pharmacological effect. Similarly, in the peritoneal exudate model, this dose significantly reduced leukocyte and neutrophil migration, although to a lesser extent compared to the 100 and 200 mg/kg doses. Thus, the 50 mg/kg dose can be considered the lowest effective dose evaluated, producing a significant, but not maximal response.

## 3. Discussion

LC-MS/MS analysis allowed the detection of 39 relevant peaks (Table 1) throughout the 60 min chromatographic run, representing different classes of phenolic compounds. Identification was performed by comparing published reference data and by isolating the compounds.

Six of these peaks (**1**, **3**, **5**, **6**, **7**, and **9**) were attributed to phenolic glycosides, showing fragmentation patterns characteristic of sugar unit loss, with neutral losses of −162 Da (hexoses), −132 Da (pentoses), or −294 Da, corresponding to the elimination of a hexose-pentose disaccharide [36]. The identified aglycones included gallic acid, gentisic acid, vanillic acid, and syringic acid, evidenced by the characteristic base ions at MS^2^ spectra formed after sugar cleavage, observed at *m*/*z* 169 ([M−162−H]^−^), 153 ([M−162−H]^−^), 167 ([M−294−H]^−^ and 197 ([M−294−H]^−^), respectively; a disaccharide loss is also shown in compound **6** and **9** [17,18,19,20,22].

Ellagic acid derivatives were detected at peaks **19**, **20**, **22**, and **25**, based on the analysis of MS^2^ spectra, which showed characteristic fragment ions at *m*/*z* 315, attributed to methyl ellagic acid, and at *m*/*z* 328, corresponding to dimethyl ellagic acid. Compounds **19** and **20** presented deprotonated molecular ions [M−H]^−^ at *m*/*z* 477 and 447, respectively. Both exhibited similar fragmentation profiles, differing mainly in the sugar unit attached to the aglycone, being a hexose in compound **19** and a pentose in compound **20**. In the MS^2^ spectra, the ion at m/z 315, related to methyl ellagic acid, showed an additional fragment at m/z 299, corresponding to the loss of a methyl group (−CH_3_). Similarly, for dimethyl ellagic acid, the sequential loss of two methyl groups was observed, evidenced by the ions at *m*/*z* 314 and 299, confirming the fragmentation pattern expected for these derivatives [26,32,33,34,35].

Compounds **2**, **4**, **8**, and **10**–**16** were assigned to the class of hydrolyzable tannins, based on the presence of characteristic fragments of this class in the MS^2^/MS^3^ spectra. In general, all the mentioned compounds showed fragmentation patterns compatible with hydrolyzable tannins, with neutral losses corresponding to sugar losses, galoyl ([M−H−152]^−^), gallic acid ([M−H−170]^−^), and HHDP ([M−H−302]^−^) groups were observed. However, compound **11**, assigned to chebulanin, differed by containing a unit derived from chebulinic acid (chebuloyl group), evidenced by the characteristic loss of 320 Da, resulting in the formation of the fragment ion at *m*/*z* 319, typical of this structural subclass [37]. Hydrolyzable tannins are widely distributed throughout various organs of the Fabaceae family, such as seeds, leaves, stems, bark, branches, and fruits. Interestingly, in the LC–MS/MS analysis performed in this study, no condensed tannins were identified, despite this class being generally regarded as more abundant in plants than hydrolyzable tannins [38,39].

**Figure 5:**
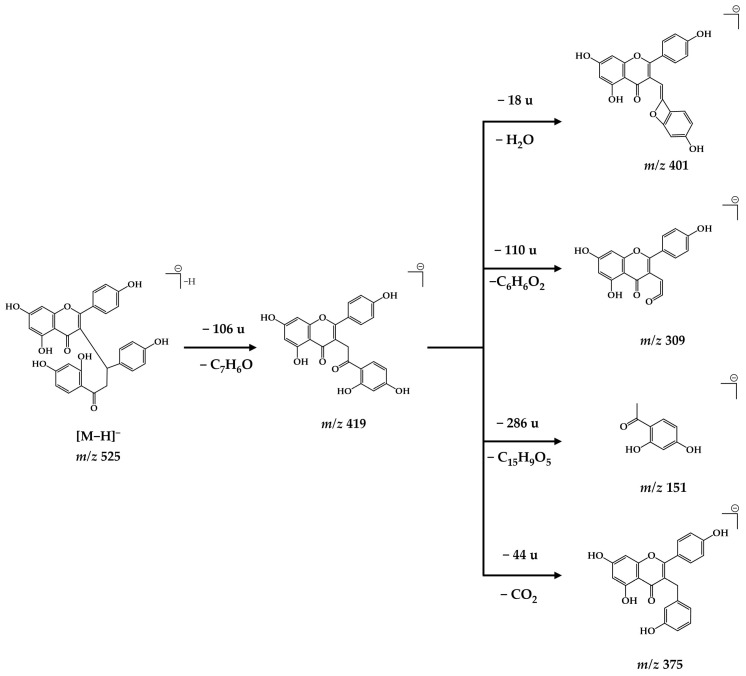
Proposed structures for the caesalpinioflavone (**27**) fragments consecutive to small neutral losses.

Peaks **23**, **24**, and **26**–**39** were assigned to biflavonoids, metabolites frequently reported in the Caesalpinioideae subfamily [40]. Compound **27** was identified after isolation, and its structural characterization by NMR allowed correlation with the corresponding mass spectrum. In LC–MS, it presented a deprotonated ion at *m*/*z* 525.1206 [M−H]^−^, and high-resolution mass spectrometry allowed the proposition of the molecular formula C_30_H_22_O_9_ (calculated for the neutral ion), confirming its identity. The proposed fragmentation of caesalpinioflavone can be seen in Figure 5.

The fragmentation of caesalpinioflavone leads to the formation of the diagnostic ion *m*/*z* 419, generated from the precursor *m*/*z* 525 [M−H]^−^ through a 1,2-aryl migration of the apigenin-derived unit (from Cβ to Cα of dihydrochalcone unity), followed by retro-Michael cleavage with neutral elimination of a *p*-quinonemethide group (106 Da). From this intermediate, three fragments are observed in the MS^3^ spectra: the *m*/*z* 401 ion, resulting from dehydration (loss of 18 Da), probably associated with intramolecular rearrangement involving the 2′ hydroxyl group of chalcone and the adjacent carbonyl group; the *m*/*z* 375 ion, formed by a neutral loss of 44 Da, is attributed to an α-cleavage of the side-chain carbonyl group with electronic rearrangement and increased conjugation of the flavonoid system [41]. The base peak *m*/*z* 309 results from the elimination of the A ring of chalcone (110 Da). All the fragments derived from the *m*/*z* 419 ion, except for *m*/*z* 309, exhibit low relative intensity in the mass spectrum, indicating that formation of the *m*/*z* 309 ion is energetically favored. Its high intensity is consistent with its greater stability, resulting from the extensive delocalization of the negative charge through the conjugated π system.

The methoxylated derivatives of caesalpinioflavone (**31**, **33-35**) are inferred by the 15 Da increase in the mass of the deprotonated ion, observed at *m*/*z* 539 [M−H]^−^, compared to the non-methoxylated compound, as well as by the molecular formula C_31_H_23_O_9_ determined by high-resolution mass spectrometry [42]. The preservation of the neutral losses of 106 Da, attributed to the elimination of the *p*-quinonemethide group, and of 110 Da, characteristic of the elimination of the resorcinol unit from ring A of the chalcone, indicates that the fragmentation pattern of the chalcone moiety remains unchanged. This behavior suggests that the methoxyl group is located in the flavone unit or in a position on ring A of the chalcone that does not interfere with the diagnostic cleavages observed.

The unknown biflavonoids (**26**, **28-30**, **32**, **36** and **37**) could not be fully annotated due to the absence of fragmentation patterns described in the literature for comparison. The preliminary assignment to this class was based on the molecular formula determined by high-resolution mass spectrometry, elution in a chromatographic region characteristically rich in biflavonoids, and the similarity of the fragmentation profile with compounds previously annotated as belonging to this class [43]. Thus, the present data broaden the phytochemical knowledge of the species and genus, and provide a basis for guiding future investigations aimed at isolating and structurally elucidating new biflavonoids, potentially novel for the species.

The preparations used traditionally are frequently administered orally, in the form of infusions, decoctions, or extracts. Despite their widespread traditional use, medicinal plant preparations require scientific validation regarding both efficacy and safety, including integrated toxicological assessments [44,45]. In this context, considering that the oral toxicity of *C. pluviosum* had not yet been investigated, we first evaluated the acute oral toxicity of the ethanolic extract of the stem bark of this species, which is used in traditional medicine [7].

Since no mortality was observed at the limit dose of 2000 mg/kg, the median lethal dose (LD_50_) could not be experimentally determined and was therefore considered to exceed this value. According to the guidelines of the OECD for acute oral toxicity testing, the limit dose of 2000 mg/kg is commonly employed to assess substances with low expected toxicity [44]. Thus, the LD_50_ of CEECP indicates low acute oral toxicity under the conditions tested. Based on standard body surface area conversion factors, this dose corresponds to a human equivalent dose of approximately 162 mg/kg, suggesting a relatively wide margin of safety for oral exposure [46].

Hematological analysis is an important tool for assessing the health status of animals, since the hematopoietic system is highly sensitive to chemical compounds, reflecting the physiological, nutritional, and pathological conditions of the organism. Therefore, measuring these parameters allows for the identification of possible alterations or adverse effects on blood constituents, and aids in investigating the biological effects of chemical compounds or plant extracts on the blood system [47,48].

The evaluation of biochemical parameters, especially those related to liver and kidney function, such as creatinine, urea, ALT, AST, alkaline phosphatase, and GGT, demonstrated that CEECP did not promote significant differences between the treated groups and the control group (*p* > 0.05). In general, acute toxicity assay indicated that the *C. pluviosum* extract exhibited a favorable safety profile under the evaluated conditions, with classification in Category 5 of the Globally Harmonized System (GHS), due to the absence of mortality and adverse clinical signs at the 2000 mg/kg dose. Importantly, the lack of significant alterations in hematological and biochemical parameters reinforces the absence of systemic toxicity under the experimental conditions. These findings suggest that the CEECP does not interfere with key physiological processes, particularly those related to hepatic and renal function, which are commonly affected by xenobiotics.

Toxicological assessment is particularly important, since differences in safety profiles can occur even between species belonging to the same family and subfamily. An example is the leguminous species *Albizia coriaria* Welw. ex Oliv., a medicinal tree whose stem bark is traditionally used to treat flu, coughs, fever, inflammation, pain and snake bites. Studies with the ethanolic extract of the stem bark of this species in rats have reported several behavioral, physiological and biochemical changes associated with exposure to the extract, such as piloerection, hyperventilation, lethargy, and significant and progressive increases in body weight, erythrocyte count, alkaline phosphatase and AST levels; also, it showed an estimated LD_50_ close to 2000 mg/kg; contrary to the evidence in our study, where the same dose did not cause mortality or adverse clinical signs, indicating that it is a safe dose [49].

Further studies are needed to characterize the toxicological profile of species of the genus *Cenostigma*, especially considering their use in traditional medicine and the fact that some exhibit significant toxic effects. In this context, *Cenostigma pyramidale* stands out for having well-established toxicological effects in livestock, such as sheep and goats. In these animals, significant reproductive and teratogenic effects are described, including early embryonic loss and abortions, especially when the plant is consumed by females in the early stages of gestation. Notably, the toxic effects persist even in dried leaves, leading to increased cases of poisoning during dry periods. On the other hand, there is no evidence of significant toxicity in males, and a positive effect on body development has even been reported [50].

Also belonging to the genus, the species *Cenostigma macrophyllum* has been used in folk medicine for the treatment of stomach and intestinal diseases, commonly as decoctions or infusions prepared from stem bark, leaves and flowers. Its leaf extract was administered orally to female rats at a dose of 500 mg/kg, and no estrogenic activity or histopathological changes in the heart, kidneys, and liver of pregnant rats were observed. However, it interfered with the estrous cycle, reducing the number of estrus cycles and increasing their duration, indicating the need for caution in its use in women of reproductive age [51,52]. These findings highlight the complexity of the toxicological profile within the genus and reinforce the need for specific evaluations for each species.

Although no signs of acute toxicity were observed following a single oral administration at the limit dose over a 14-day observation period, our study is limited to acute exposure and does not provide information on potential subchronic or chronic effects. Therefore, additional studies involving repeated-dose toxicity and mechanistic approaches are necessary to better characterize the toxicological profile of the CEECP.

The perception of greater safety associated with medicinal plants, when compared to synthetic drugs, has contributed to the increased use of phytoderivatives in the treatment of various health conditions. However, the traditional and sometimes indiscriminate use of these products raises concerns about their potential to cause adverse effects, especially regarding toxicity. Despite their widespread traditional use, medicinal plants require scientific validation regarding both efficacy and safety, including integrated toxicological assessments. Given that plant extracts are complex mixtures of bioactive compounds, they carry the potential to induce mutagenic and genotoxic effects, including chromatid breaks and fragmentations, gap formation, sister chromatid exchanges and fusions, as well as structural alterations in DNA [53,54].

The mutagenic potential of CEECP was evaluated in mice using micronucleus and comet assays, revealing no evidence of mutagenicity or myelotoxicity at the tested dose. These experimental results corroborate previous studies demonstrating the absence of mutagenic activity in the flowers and leaves of *Cenostigma pluviosum* var. *peltophoroides* (basionym: *Caesalpinia peltophoroides* Benth.) and indicate a potential antimutagenic effect. *C. peltophoroides* fractions significantly reduced the frequency of micronuclei in bone marrow erythroblasts compared to the positive control treated with cyclophosphamide. Notably, the ethyl acetate fraction of the flower achieved a 60.1% reduction, and the methanolic fraction of the leaf achieved a 55% reduction. No fraction increased the frequency of micronuclei compared to the negative control, indicating the absence of intrinsic mutagenicity. Collectively, statistically significant reductions exceeding 48% reinforce the potential of these fractions to protect against genetic damage induced by mutagenic agents [55].

In the comet assay, CEECP did not increase DNA damage, showing results comparable to the negative control, while doxorubicin significantly elevated damaged cells and damage scores. CEECP did not induce micronucleus formation or DNA damage in the comet assay, indicating no detectable genotoxic effects under the tested conditions [53,54]. In contrast, studies with *Cenostigma gardnerianum* stem bark have demonstrated distinct behavior, in which the ethanolic extract of the bark showed a high frequency of micronuclei in the *Allium cepa* test, especially at concentrations from 2 mg/mL, indicating genotoxic potential and the ability to induce damage to cellular DNA. This divergence in results may be related to phytochemical differences between the species, as well as the experimental models employed (plant vs. mammal) [56]. Thus, while *C. gardnerianum* shows evidence of genotoxicity under specific conditions, *C. pluviosum* demonstrates a more favorable genotoxic safety profile, underscoring its potential for safe pharmacological applications.

The type of solvent used in extraction can significantly influence the toxicological profile of the resulting extract. In general, aqueous extracts tend to have lower genotoxic potential compared to methanolic extracts. Due to its intermediate polarity, ethanol efficiently extracts a wide spectrum of phytochemicals, resulting in more chemically complex extracts, which can impact their biological and toxicological profile. Therefore, the evaluation of possible genotoxic and mutagenic effects of ethanolic extracts becomes particularly relevant, considering their wide use and efficiency in the extraction of bioactive metabolites [57].

The CEECP is characterized as a rich source of phenolic compounds; these constituents may underlie both the observed pharmacological effects and their potential protective properties; they are frequently described in the literature for their antioxidant capacity and their modulation of mechanisms involved in maintaining DNA integrity [58,59]. Our study represents an important step in characterizing the genotoxic profile of *C. pluviosum*, providing preliminary evidence of safety under controlled experimental conditions. These findings establish a scientific basis for future, more comprehensive investigations, including long-term exposure studies, additional experimental models, and mechanistic analyses, contributing to the consolidation of knowledge about the genotoxic safety of the species.

Considering the carrageenan-induced paw edema assay, the tested doses of 50, 100, and 200 mg/kg correspond to approximate human equivalent doses of 4, 8, and 16 mg/kg, respectively, based on standard body surface area conversion factors. These human equivalent doses, together with the favorable toxicological profile observed, suggest a relatively wide margin of safety for potential oral use [46].

The anti-inflammatory effects observed for CEECP are consistent with those described for species related to the same genus, such as *Cenostigma macrophyllum*. In that study, the extract promoted a significant reduction in formalin-induced paw edema, with notable percentage inhibitions at 100 mg/kg, in addition to decreasing carrageenan-induced neutrophil migration, pointing to an effect on the cellular phase of inflammation. Similarly, CEECP also demonstrated relevant anti-inflammatory activity in the present study, evidenced by the significant reduction in the inflammatory parameters evaluated. Both species share anti-inflammatory activity and *C. macrophyllum* also demonstrated a similar phytochemical profile to *C. pluviosum*, comprising ellagic acid, gallic acid derivatives and biflavonoids [51]. Taken together, the results are consistent with the pharmacological and phytochemical similarities between phylogenetically related species, and suggest that shared bioactive compounds, such as phenolic compounds, may be responsible for the observed effects.

Previous studies with *C. pluviosum* leaf extract demonstrated that it significantly inhibited prostaglandin E_2_ (PGE_2_) production in ex vivo assays, in addition to showing notable anti-inflammatory activity in a rat ear edema model, with 70.90% inhibition, a value statistically superior to the negative control and comparable to that of dexamethasone (64.10%). These results indicate that the extract contains bioactive compounds capable of acting at different levels of the inflammatory response, both in ex vivo experimental systems and in vivo models. According to the phytochemical characterization of the stem bark carried out in this study, a chemical similarity is observed in relation to the leaves, as both contain ellagic acid, gallic acid and their derivatives, in addition to biflavonoids. However, they differ in that the leaves exhibit a broader chemical profile, with the presence of condensed tannins, flavonoid monomers and their glycosides, as well as nitrogenous compounds, which were not detected in the stem bark extract [60].

As evidenced by LC-MS/MS analysis, the remarkable anti-inflammatory activity of CEECP can be attributed to its phytocomplex comprising various classes of polyphenols, including tannins, flavonoids, ellagic acid derivatives and phenolic acid heterosides. Several reports in the literature show that tannins have anti-inflammatory, analgesic, immunomodulatory, antinociceptive, and antipyretic effects [61].

Polyphenols, like tannins, can alleviate inflammation by modulating inflammatory mediators, inhibiting inflammatory enzymes, and suppressing pro-inflammatory transcription factors. These compounds can inhibit enzymes such as cyclooxygenases (COX-1 and COX-2), reducing the production of prostaglandins, especially PGE_2_; suppress the expression of pro-inflammatory cytokines, such as TNF-α, IL-6, and IL-8, as well as decreasing the activation of nuclear factor kappa B (NF-κB), an important regulator of the expression of genes involved in the inflammatory response; and reduce nitric oxide (NO) production by inhibiting the iNOS enzyme. Anti-inflammatory activity through neutralization of free radicals and reactive oxygen species (ROS), as well as inhibition of ROS-producing enzymes such as xanthine oxidase and NADPH oxidase, has also been widely reported [62,63,64].

The injection of carrageenan into the rat paw induces edema through the release of inflammatory mediators in two phases. The early phase, occurring within the first two hours, involves the release of vasoactive mediators such as histamine, serotonin, and bradykinin, leading to increased permeability and vasodilation. The late phase, extending from approximately the second to the fifth hour, involves the production of additional inflammatory mediators and further progression of the inflammatory response [65,66,67].

The paw edema and peritonitis assays complement each other in evaluating the different phases of acute inflammation. While the carrageenan-induced paw edema assay assesses the extract’s ability to modulate the mediators involved in the initial phases of inflammation, as evidenced by the significant reduction in edema observed in this study, the peritonitis model reflects the cellular phase by evaluating leukocyte recruitment to the peritoneal cavity. In this context, the results suggest that CEECP exerts anti-inflammatory activity, possibly through modulation of vasoactive mediators from the early stages of the inflammation. Consequently, by acting in the initial phases of inflammation, the extract reduces the production of pro-inflammatory cytokines such as TNF-α and IL-1β, leading to decreased leukocyte migration [68,69].

NF-κB stands out as one of the main regulators of the inflammatory response, transducing signals from the cytoplasm to the nucleus and regulating the expression of several genes involved in the formation of pro-inflammatory cytokines (TNF-α, IL-1, and IL-6), chemokines, and cell adhesion molecules [70]. Several molecules reported in the literature were also detected in our LC–MS/MS analysis and have been associated with the modulation of pro-inflammatory mediators. Among them, the hydrolyzable tannins identified in this study, corilagin [71,72], chebulanin [73], chebulagic acid [74] and pedunculagin [75], have been shown to inhibit NF-κB and/or modulate its signaling pathways, thereby reducing the expression of pro-inflammatory mediators. The reduction in TNF-α and IL-1β levels suggests a possible modulation of upstream inflammatory signaling pathways, potentially involving NF-κB activation, although this mechanism was not directly investigated in the present study.

These anti-inflammatory properties of ellagitannins are supported by experimental data in a variety of models. Corilagin has shown in vivo efficacy in treating cholestasis at 40 mg/kg, lowering oxidative stress indicators and reducing NF-κB activation, evidenced by decreased nuclear translocation of the p65 subunit. Chebulanin exhibited anti-arthritic effects at 80 mg/kg and inhibited NF-κB and MAPK signaling pathways, as demonstrated by reduced phosphorylation of IκBα and NF-κB p65, as well as suppression of MAPK components such as p38 and JNK. Interestingly, less polar and more structurally complex tannins like chebulagic acid and pedunculagin demonstrated greater activity at lower concentrations (5–25 µM), inhibiting NF-κB and MAPK activation in cellular models and suppressing pro-inflammatory mediators like NO, PGE_2_, TNF-α, and IL-6. These effects were associated with decreased phosphorylation of IκBα, reduced nuclear levels of NF-κB subunits (p65 and p50), and inhibition of MAPK signaling proteins, including p38, JNK, and ERK [71,72,73,74,75].

The metabolism of ellagitannins and ellagic acid derivatives involves the hydrolysis of the sugar fraction of these molecules, releasing ellagic acid, which is subsequently converted by the gut microbiota into urolithins. These metabolites have been extensively studied for their anti-inflammatory effects, acting on the modulation of inflammatory genes and proteins, such as inducible nitric oxide synthase (iNOS, COX-2, PTGES, and NF-κB), as well as prostaglandins and pro-inflammatory cytokines involved in the regulation of the inflammatory response and associated processes, such as fibroblast migration and monocyte adhesion [76,77].

Caesalpinioflavone, identified by mass spectrometry and subsequently isolated in this study, has been previously reported to exhibit anti-inflammatory activity. The compound promoted a 69.23% reduction in ear edema at a dose of 0.5 mg/kg, greater than that of the reference drugs indomethacin and dexamethasone (43.38% and 56.00%, respectively), and may therefore play a significant role in the overall anti-inflammatory effect of the extract [78,79].

In summary, the synergistic action of the phenolic compounds identified in CEECP likely underlies its significant anti-inflammatory activity and may potentiate its pharmacological effects. Finally, the safety profile demonstrated in toxicological assays supports CEECP as a promising source of phytocompounds with pharmacological applications.

## 4. Materials and Methods

### 4.1. Plant Material

The stem bark *of Cenostigma pluviosum* var. *peltophoroides* was collected in May 2018 from the garden of the Pharmaceutical Technology Laboratory, located on campus I of the Federal University of Paraíba, João Pessoa (7°8′28.0104″ S and 34°50′47.958″ W), Paraíba. The specimens were identified, and an exsiccate (code Agra et al. 7040) is deposited in the Herbarium Prof. Lauro Pires Xavier (JPB). The study is registered in the Sistema Nacional de Gestão do Patrimônio Genético e do Conhecimento Tradicional Associado (SisGen) under code A339C6A.

### 4.2. Extraction and Chemical Characterization of the CEECP by LC-MS^n^

The stem barks were dried at 40 °C and 14 kg of stem bark powder was obtained after pulverization. The powder was macerated in 95% EtOH, using a stainless steel container, for 72 h, and the process was repeated three times. The extractive solution was concentrated in a rotary evaporator at a temperature of 45–50 °C, obtaining the crude ethanolic extract (377.1 g).

HPLC-MS analyses were performed using a Shimadzu (Shimadzu Corporation, Kyoto, Japan) analytical high-performance liquid chromatograph equipped with a SIL-20A HT autoinjector, two LC-20AD pumps, a DGU-20A5 on-line degasser, a SPD-M20A UV-VIS detector, and a CBM-20A control system. A Kromasil C-18 5 μm 100 Å, 250 mm × 4.6 mm (Kromasil^®^, Nouryon, Bohus, Sweden) analytical column was connected to the system. For the liquid chromatography system, acidified ultrapure water (0.1% formic acid) and chromatographic-grade methanol served as mobile phases A and B, respectively. In the elution method, the concentration of mobile phase B was gradually increased from 5% to 100% over a 60 min run, with a flow rate of 0.6 mL/min. Column temperature was maintained at room temperature (20 °C). For low-resolution acquisition, the chromatograph was coupled to a Bruker Ion-Trap Amazon X (Bruker Daltonics, Billerica, MA, USA) mass spectrometer with an electrospray ionization (ESI) source. The analysis parameters were: capillary 3.5 kV, ESI operating in negative mode, end plate offset 500 V, nebulizer 4 psi, dry gas (N_2_) with a flow rate of 8 L/min and temperature of 200°C. CID fragmentation was achieved in auto MS/MS mode using the advanced resolution mode for MS and MS/MS mode. The spectra (*m*/*z* 50–1500) were recorded every 2 s. For the analysis, a sample of 1 mg of the crude ethanolic extract of *C. pluviosum* stem bark (CEECP) was dissolved in methanol to obtain a 1 mg·mL^−1^ solution. The solution was filtered through a 0.45 μm PVDF membrane, and 10 μL was injected into the chromatographic system.

Subsequently, the same sample prepared for low-resolution analysis was reinjected into a high-resolution mass spectrometer, BRUKER^®^ micrOTOF II (Bruker Daltonics, Billerica, MA, USA), with an electrospray ionization source, operating in negative mode. For high-resolution HPLC-ESI-MS analyses, the same column and the same chromatographic conditions were used as described above. The micrOTOF II spectrometer analysis parameters were 4.0 kV capillary, a 500 V end plate offset, a 4.0 bar nebulizer, a dry gas (N_2_) flow rate of 8.0 L/min, and a temperature of 200 °C. Spectra (*m*/*z* 50–1000) were recorded every 2.0 s. Sodium formate was used as the internal calibrator during the chromatographic run.

Compounds were annotated based on the ions of the deprotonated molecule, fragmentation patterns (MS^2^ and MS^3^), and corresponding data from scientific literature. Details regarding the isolation and structural characterization of caesalpinioflavone can be found in the Supplementary Material section.

### 4.3. In Vivo Toxicity Experiments

#### 4.3.1. Ethical Procedures and Vivarium Conditions

The experimental protocols were approved by the Ethics Committee on the Use of Animals (CEUA) of the UNIFACISA University Center, under approval number 03.0001.2024/05.2024, in accordance with the standards established by the National Council for the Control of Animal Experimentation (CONCEA). For the pharmacological assays, adult Swiss albino mice (*Mus musculus*), weighing between 28 and 31 g and aged between 50 and 54 days were used, with animals of both sexes employed in the acute oral toxicity test. The animals were kept under standard laboratory conditions, with a 12 h light/dark cycle, controlled temperature (22 ± 2 °C) and relative humidity between 50 and 55%, receiving filtered water and standard rodent feed ad libitum. Euthanasia was performed by intraperitoneal administration of a hyperdose of 10% ketamine (300 mg/kg) combined with 2% xylazine (30 mg/kg), in accordance with the CONCEA Euthanasia Practice Guidelines (2018).

#### 4.3.2. Acute Toxicity Assay

The acute toxicity of the ethanolic extract of the stem bark of *Cenostigma pluviosum* var. *peltophoroides* (Benth.) Gagnon & G.P.Lewis (CEECP) was evaluated according to the instructions of the Organization for Economic Co-operation and Development (OECD). Female mice were separated into two groups (*n* = 5 for each group) that received *C. pluviosum* var. *peltophoroides* (2000 mg/kg) or 0.9% saline by gavage in a single oral dose (100 µL/10 g bw). Behavioral changes were evaluated during the first 4 h according to the parameters described by Malone (1983) [80] and Almeida et al. (1999) [81]. Weight gain and water and food consumption were measured daily until the 14th day. On the 15th day after the beginning of treatment and fasting for 6 h, the animals were anesthetized with ketamine 100 mg/kg and xylazine 5 mg/kg solution, and blood was collected for biochemical and hematological analyses. The LD_50_ value was determined according to the method described in OECD Guidelines 423 [82].

#### 4.3.3. Biochemical and Hematological Analysis

At the end of toxicity assays, blood was collected from the animals and the following biochemical parameters were evaluated: total protein, albumin, alanine aminotransferase (ALT), aspartate aminotransferase (AST), alkaline phosphatase, gamma-glutamyl transferase (GGT), urea and creatinine, using specific kits (Labtest Diagnóstica, Lagoa Santa, Brazil) and a COBAS Mira Plus analyzer (Roche Diagnostics Systems, Basel, Switzerland). Hematological analysis was performed using an automatic analyzer (Counter-ABC Vet Animal Blood, Montpellier, France) and optical microscopy; the parameters evaluated were: erythrocytes, hemoglobin, hematocrit, mean corpuscular volume (MCV), mean corpuscular hemoglobin (MCH), mean corpuscular hemoglobin concentration (MCHC) and total and differentiated leukocyte analysis.

#### 4.3.4. Genotoxicity and Mutagenicity Assessment

For the comet assay, an aliquot (10 µL) of blood from each animal was mixed with 0.5% agarose at 36.5 °C on microscope slides. The slides were covered with coverslips at 4 °C for 30 min. Then, the coverslips were removed and the slides were immersed for 1 h in lysis solution [2.0 M NaCl, 100 mM EDTA, 10 mM Tris, 0.2 M NaOH, 2% (*w*/*v*) sodium lauryl sarcosine, 0.5% (*v*/*v*) Triton X-100, and 1% (*v*/*v*) dimethyl sulfoxide] at 4 °C, protected from light. Subsequently, an electrophoresis run was performed at 4 °C for 20 min at 280 mA and 25 V. The slides were fixed in 100% ethanol for 30 min and covered with 30 mL of ethidium bromide staining solution (30 mg/mL) and coverslips. The evaluation was performed under a fluorescence microscope (400×) and 600 cells were evaluated for the presence of nucleoids and classified according to the tail size into the following classes: 0—no tail; 1—tail smaller than the diameter of the head (nucleus); 2—tail length measuring 1–2 times the diameter of the head; 3—tail length measuring more than twice the diameter of the head [83].

To evaluate the genotoxic profile of CEECP treatment, the micronucleus test was used in mouse erythrocytes based on the method described by Oliveira et al. with minor modifications [84]. Briefly, groups of male mice (*n* = 5) were treated with saline (negative control) or CEECP (2000 mg/kg) orally in a single dose. 24 h after treatments, aliquots (10 µL) of peripheral blood (tail) were applied to slides previously stained with acridine orange and analyzed under a fluorescence microscope. A total of three slides per animal were prepared and the micronucleus frequency was determined after counting 2000 erythrocytes per slide.

### 4.4. Anti-Inflammatory Activity

The doses of 50, 100, and 200 mg/kg were selected for the anti-inflammatory assays based on previous studies evaluating the pharmacological effects of species from the genus *Cenostigma*, in which this range is commonly employed in in vivo models [51,85,86]. Additionally, the selection of these doses was supported by the results of the acute oral toxicity test. Therefore, the chosen doses fall within a safe range for pharmacological evaluation while allowing the assessment of dose-dependent effects.

#### 4.4.1. Carrageenan-Induced Paw Edema

Male mice were divided into five groups (*n* = 6) and treated with CEECP (50, 100 or 200 mg/kg *p.o.*), saline (0.9% *p.o.*, orally) or indomethacin (20 mg/kg, positive control, intraperitoneally), 30 min before edema induction. Subsequently, paw edema was induced by injecting 15 μL of 2% (*w*/*v*) carrageenan into the subplantar region of the right paw. For comparison, the left paw received 15 μL of saline solution. Paw edema was evaluated by measuring hind paw thickness using a digital caliper at 1, 2, 3, and 4 h after carrageenan injection [87]. The percentage change in hind paw thickness (%) was determined using the following formula:

(1) $$\mathrm{Percentage}\;\mathrm{change}\;\mathrm{in}\;\mathrm{hind}\;\mathrm{paw}\;\mathrm{thickness}=\left(\frac{\mathrm{final}\;\mathrm{hind}\;\mathrm{paw}\;\mathrm{thickness}-\mathrm{initial}\;\mathrm{hind}\;\mathrm{paw}\;\mathrm{thickness}}{\mathrm{final}\;\mathrm{hind}\;\mathrm{paw}\;\mathrm{thickness}}\right)\times 100$$

#### 4.4.2. Peritonitis

Male mice were divided into five groups (*n* = 6) and treated with CEECP (50, 100, or 200 mg/kg *p.o.*), saline (0.9% *p.o.*), or indomethacin (20 mg/kg i.p.) 30 min before the start of the assay. The animals received an intraperitoneal injection of 1% carrageenan. After 4 h, the animals were sacrificed and 2 mL of heparinized PBS was injected into the peritoneal cavity; the exudate was then immediately collected. The leukocyte count of the peritoneal lavage was expressed as leukocytes/mL [88,89]. The percentage of leukocytes inhibition was calculated using the following formula:

(2) % Leukocyte inhibition = (1 − T/C) × 100

where T represents the number of leukocytes in the treated group (extract or reference drug), and C represents the number of leukocytes in the control group. Cytokine levels in the peritoneal fluid were quantified using a bead-based multiplex immunoassay (Milliplex MAP 7-plex kit, Millipore, Merck, Darmstadt, Germany), according to the manufacturer’s instructions [90].

### 4.5. Statistical Analysis

The data obtained were analyzed in GraphPad Prism^®^ version 8.0 and expressed as mean values with standard deviation (±SD). Statistically significant differences were calculated using one-way analysis of variance (ANOVA) followed by Bonferroni or Dunnett’s test (when necessary). Values were considered significantly different at *p* < 0.05.

## 5. Conclusions

The chemical profile of the ethanolic extract of the stem bark of *Cenostigma pluviosum* var. *peltophoroides* revealed a predominance of phenolic compounds across different classes, including biflavonoids, hydrolyzable tannins, lignans, and phenolic acids derivatives. In vivo toxicity test showed a safe profile of the CEECP at a dose of 2000 mg/kg, with no evidence of genotoxicity or mutagenicity. Notably, this is the first report describing the toxicological safety of the extract of *Cenostigma pluviosum* var. *peltophoroides*. In pharmacological assays, CEECP showed dose-dependent anti-inflammatory activity, with an anti-edematogenic effect already observed at a dose of 50 mg/kg and more pronounced responses at doses of 100 and 200 mg/kg, in the first hour of the experiment. Furthermore, the CEECP was able to significantly reduce the migration of total leukocytes and neutrophils, as well as decrease the levels of the pro-inflammatory cytokines TNF-α and IL-1β.

In combination, these results demonstrate the anti-inflammatory potential of *C. pluviosum* and confirm the safety profile of the CEECP at the doses evaluated. Moreover, the study opens perspectives for future investigations aimed at elucidating the mechanisms of action involved, identifying new secondary metabolites present in the species and supporting the potential development of botanical extracts for phytotherapeutic applications, including topical or oral formulations, as new anti-inflammatory agents.

## Figures and Tables

**Table 1:** Phytochemical profile of the crude ethanolic extract of the stem bark of *Cenostigma pluviosum* by HPLC–ESI–MS^n^.

Peak	R.T. (min)	[M−H]^−^ *m*/*z*	Molecular Formula	Calculated	Error (ppm)	MS^2^/MS^3^	Annotation	Ref.
**1**	11.1	331.0677	C_13_H_15_O_10_	331.0671	−1.9	MS^2^ [331]: 313; 271; 241; 211; 193; **169**; 125/MS^3^ [331 ➔ 169]: **125**, 97	Galloyl-O-hexoside	[17]
**2**	16.9	483.0782	C_20_H_19_O_14_	483.0780	−0.4	MS^2^ [483]: **331**; 313; 271; 169/MS^3^ [483 ➔ 331]: **313**; 271; 241; 193; 169; 151; 125	Digalloyl-O-hexoside isomer	[18]
**3**	17.0	315.0725	C_13_H_15_O_9_	315.0722	−1.1	MS^2^ [315]: 225; 179; **153**; 108/MS^3^ [315 ➔ 153]: **151**; 109	Gentisic acid-O-hexoside	[19]
**4**	17.4	495.0790	C_21_H_19_O_14_	495.0780	−2.0	MS^2^ [495]: **343**; 325; 289; 169/MS^3^ [495 ➔ 343]: 191; **169**; 125	Digalloyl quinic acid	[20]
**5**	17.5	329.0885	C_14_H_17_O_9_	329.0878	−2.2	MS^2^ [329]: **167**/MS^3^ [329 ➔ 167]: **152**; 123; 108	Vanillic acid-O-hexoside	[18]
**6**	17.8	461.1296	C_19_H_25_O_13_	461.1301	0.9	MS^2^ [461]: 461; 329; 293; **167**; 152;/MS^3^ [461 ➔ 167]: **152**; 123; 108	Vanillic acid-O-hexoside-pentoside	[21]
**7**	19.8	359.0995	C_15_H_19_O_10_	359.0984	−3.2	MS^2^ [359]: **197**/MS^3^ [359 ➔ 197]: 182; 153; **138**	Syringic acid-O-hexoside	[22]
**8**	20.4	483.0782	C_20_H_19_O_14_	483.0780	−0.4	MS^2^ [483]: 423, 331, **313**, 271, 169/MS^3^ [483 ➔ 313]: 285; 242; 179; **169**	Digalloyl-O-hexoside isomer	[23]
**9**	20.4	491.1429	C_20_H_27_O_14_	491.1406	−4.6	MS^2^ [491]: 370; **197**/MS^3^ [491 ➔ 197]: 182; **138**; 121	Syringic acid-O-hexoside-pentoside	[21,22]
**10**	22.5	801.0831	C_34_H_25_O_23_	801.0792	−4.9	MS^2^ [801]: **757**; 631/MS^3^ [801 ➔ 757]: 713; 631; **613**; 603; 435; 273	Mallotinic acid	[24]
**11**	25.2	651.0828	C_27_H_23_O_19_	651.0839	1.7	MS^2^ [651]: **633**; 607; 481; 463; 319; 275; 231; 205/MS^3^ [651 ➔ 633]: 589; 545; **463**; 331; 319; 275, 257; 231	Galloyl-chebuloyl-hexose (Chebulanin)	[25]
**12**	26.0	633.0755	C_27_H_21_O_18_	633.0733	−3.5	MS^2^ [633]: 589; 463; 419; **301**; 275; 245/MS^3^ [633 ➔ 301]:	Galloyl-HHDP-hexose (Corilagin)	[26]
**13**	28.0	953.0937	C_41_H_29_O_27_	953.0902	−3.7	MS^2^ [953]: **935**; 909; 801; 633; 463; 301/MS^3^ [953 ➔ 935]: 633; **463**; 301	Galloyl-Chebuloyl-HHDP-hexose (Chebulagic acid)	[27]
**14**	28.2	469.0050	C_21_H_9_O_13_	469.0049	−0.3	MS^2^ [469]: **425**/MS^3^ [469 ➔ 425]: **299**	Tergallic acid dilactone/valoneic acid bilactone	[28]
**15**	29.2	785.0843	C_34_H_25_O_22_	785.0843	−0.1	MS^2^ [785]: **633**; 617; 616; 483/MS^3^ [785 ➔ 633]: 589; 463; 437; 419; **301**; 275	Digalloyl-HHDP-hexose (Pedunculagin II)	[29,30]
**16**	30.4	785.0843	C_34_H_25_O_22_	785.0843	−0.1	MS^2^ [785]: 615; 465; **301**; 275; 245/MS^3^ [785 ➔ 301] **257**	Digalloyl-HHDP-hexose isomer	[29,30]
**17**	30.6	197.0453	C_9_H_9_O_5_	197.0455	1.0	MS^2^ [197]: **169**/MS^3^ [197 ➔ 169]: **125**	Ethyl gallate	[31]
**18**	31.1	521.2038	C_26_H_33_O_11_	521.2028	−1.9	MS^2^ [521]: **359**/MS^3^ [521 ➔ 359]: 344; 296	Cyclolariciresinol-O- hexoside	[18]
**19**	34.2	477.0683	C_21_H_17_O_13_	477.0675	−1.7	MS^2^ [477]: **315**, 300/MS^3^ [477 ➔ 300]: **300**, 243, 228	Methyl ellagic acid-*O*-hexoside	[32,33]
**20**	39.2	447.0570	C_20_H_15_O_12_	447.0569	−0.3	MS^2^ [477]: **315**, 300/MS^3^ [477 ➔ 300]: **300**, 243, 228	Methyl ellagic acid-*O*-pentoside	[34]
**21**	39.4	723.5044	-	-	-	MS^2^ [723]: **677**/MS^3^ [723 ➔ 677]: 659; 593; **451**; 367; 225	Unknown	-
**22**	39.9	461.0728	C_21_H_17_O_12_	461.0725	−0.6	MS^2^ [461]: 446; **328**/MS^3^ [461 ➔ 328]: **313**	Dimethyl ellagic acid-*O*-pentoside	[26,35]
**23**	43.0	523.1039	C_30_H_19_O_9_	523.1035	−0.8	MS^2^ [523]: 413; **387**; 326; 197; 169/MS^3^ [523 ➔ 387]: 293	Unknown biflavonoid	-
**24**	44.8	523.1015	C_30_H_19_O_9_	523.1035	3.7	MS^2^ [523]: 413; **387**; 326; 197; 169/MS^3^ [523 ➔ 387]: 343; 329; 293; 281; 235	Unknown biflavonoid	-
**25**	45.1	329.0303	C_16_H_9_O_8_	329.0303	−0.1	MS^2^ [329]: **314**; 299, 284; 270/MS^3^ [329 ➔ 314]: 299; 285; 271	Dimethyl ellagic acid	[34]
**26**	45.4	509.1255	C_30_H_21_O_8_	509.1242	−2.5	MS^2^ [509]: 399; **373**; 293/MS^3^ [509 ➔ 373]: 355; 279; 271; **253**; 237; 209; 185; 135	Unknown biflavonoid	-
**27**	48.4	525.1206	C_30_H_21_O_9_	525.1191	−2.9	MS^2^ [525]: **419**; 415; 309; 151/MS^3^ [525 ➔ 419]: 401; 375; **309**; 151	Caesalpinioflavone *	-
**28**	50.4	509.1252	C_30_H_21_O_8_	509.1242	−2.0	MS^2^ [509]: 399; **373**/MS^3^ [509 ➔ 373]: 305; 271; **253**; 209; 135	Unknown biflavonoid	-
**29**	50.4	537.1171	C_31_H_21_O_9_	537.1191	3.7	MS^2^ [537]: 443; 431; 427; **401**; 375/MS^3^ [537 ➔ 401]: **386**; 307; 295; 293; 281; 235; 165	Unknown biflavonoid	-
**30**	50.8	509.1245	C_30_H_21_O_8_	509.1242	−0.6	MS^2^ [509]: 399; **373**/MS^3^ [509 ➔ 373]: 305; 263; 253; **237**; 153; 135	Unknown biflavonoid	-
**31**	52.4	539.1343	C_31_H_23_O_9_	539.1348	0.9	MS^2^ [539]: **433**; 375; 323/MS^3^ [539 ➔ 433]: 415; 389; 323	Methoxylated derivative of caesalpinioflavone	-
**32**	52.8	509.1250	C_30_H_21_O_8_	509.1242	−1.7	MS^2^ [509]: 399; **373**; 363/MS^3^ [509 ➔ 373]: 355; 271; **253**; 209; 185; 135	Unknown biflavonoid	-
**33**	53.1	539.1356	C_31_H_23_O_9_	509.1348	−1.6	MS^2^ [539]: **433/** MS^3^ [539 ➔ 433]: 415, 389; **339**; 309; 295; 283; 239, 151	Methoxylated derivative of caesalpinioflavone	-
**34**	54.1	539.1340	C_31_H_23_O_9_	539.1348	1.5	MS^2^ [539]: **433**; 323/MS^3^ [539 ➔ 433]: 415; 389; **323**	Methoxylated derivative of caesalpinioflavone	-
**35**	54.8	539.1348	C_31_H_23_O_9_	539.1348	−0.1	MS^2^ [539]: 495; **429**; 308/MS^3^ [539 ➔ 429]: 308	Methoxylated derivative of caesalpinioflavone	-
**36**	54.9	551.0985	C_31_H_19_O_10_	551.0984	−0.3	MS^2^ [539]: 536; 493; 483; 455; 399; 389; **375**; 331; 307/MS^3^ [551 ➔ 375]: 331; 307	Unknown biflavonoid	-
**37**	55.6	509.1234	C_30_H_21_O_8_	509.1242	1.6	MS^2^ [509]: **399**; 373/MS^3^ [509 ➔ 399]: 357; 355; 289; 264; **263**	Unknown biflavonoid	-
**38**	57.9	553.1500	C_32_H_25_O_9_	553.1504	0.7	MS^2^ [553]: 459; 447; 429; 401; **308**/MS^3^ [553 ➔ 308]: 295; 280; 265; 252; 221	Dimethoxylated derivative of caesalpinioflavone	-
**39**	58.5	553.1500	C_32_H_25_O_9_	553.1504	0.7	MS^2^ [553]: 509; **443**; 322; 282/MS^3^ [553 ➔ 443]: 428; **322**	Dimethoxylated derivative of caesalpinioflavone	-

R.T. = Retention time. * Identified by ^1^H and ^13^C NMR.

**Table 2:** Hematological parameters of mice treated with CEECP in acute toxicity evaluation.

Parameter	Treatment	
	Control	2000 mg/kg
Erythrocytes (10^6^/mm^3^)	6.20 ± 0.52	6.49 ± 0.38
Hematocrit (%)	41.19 ± 3.45	44.51 ± 4.16
Hemoglobin (g/dL)	14.55 ± 0.79	15.03 ± 1.05
MCV (%)	42.07 ± 4.06	45.12 ± 4.31
MCH (%)	17.46 ± 1.05	18.24 ± 1.10
MCHC (%)	36.39 ± 3.07	38.17 ± 3.19
Leukocytes (10^3^/mm^3^)	8.12 ± 0.84	8.45 ± 0.75
Segmented (%)	58.40 ± 4.54	59.09 ± 3.29
Lymphocytes (%)	27.76 ± 2.34	26.01 ± 2.06
Monocytes (%)	8.48 ± 0.67	8.29 ± 0.74

MCV: mean corpuscular volume; MCH: mean corpuscular hemoglobin; MCHC: mean corpuscular hemoglobin concentration. Values represent the mean ± SD (*n* = 5/group for acute toxicity). No significant differences (*p* > 0.05) were found in comparison with control.

**Table 3:** Biochemical parameters of the blood of mice treated with *C. pluviosum* *per os*.

Parameter	Treatment	
	Control	2000 mg/kg
Creatinine (mg/dL)	4.15 ± 0.41	4.48 ± 0.39
BUN (mg/dL)	0.49 ± 0.08	0.46 ± 0.07
Albumin (g/dL)	3.59 ± 0.32	3.40 ± 0.35
Total protein (g/dL)	9.65 ± 0.67	9.72 ± 0.82
ALT (U/L)	42.44 ± 4.15	39.54 ± 4.09
AST (U/L)	39.18 ± 3.54	38.22 ± 3.65
Bilirubin	0.49 ± 0.03	0.45 ± 0.04
Alkaline phosphatase (IU/L)	8.43 ± 0.45	8.95 ± 0.67
GGT (U/L)	7.29 ± 0.32	7.81 ± 0.65
Total Cholesterol (mg/dL)	91.04 ± 5.46	95.01 ± 7.58

BUN: blood urea nitrogen; ALT: Alanine aminotransferase; AST: Aspartate aminotransferase; GGT: gamma-glutamyl transferase. Values represent the mean ± SD (*n* = 5/group for acute toxicity). No significant differences (*p* > 0.05) were found in comparison with control.

**Table 4:** Evaluation of the relative weight (g/10 g animal body weight) of mice treated daily with *C. pluviosum* extract *per os*.

Parameter	Treatment	
	Control	CEECP 2000 mg/kg
Liver	2.24 ± 0.19	2.37 ± 0.14
Kidney	0.39 ± 0.02	0.41 ± 0.03
Lung	0.29 ± 0.03	0.27 ± 0.04
Heart	0.25 ± 0.03	0.27 ± 0.03
Spleen	0.21 ± 0.03	0.19 ± 0.02

Values represent the mean ± SD (*n* = 5/group for acute toxicity). No significant differences (*p* > 0.05) were found in comparison with control.

**Table 5:** Evaluation of in vivo genotoxicity of *C. pluviosum* by determination of the number of micronucleated polychromatic erythrocytes (MNPCE) from bone marrow of mice.

Treatments	Number of MNPCE per Animal						
	M1	M2	M3	M4	M5	Mean MNPCE	PCE/NCE
Negative control	1	1	0	1	1	0.8 ± 0.1 #	1.02 ± 0.11 #
CEECP 2000 mg/kg	0	1	1	1	1	0.8 ± 0.1 #	1.09 ± 0.08 #
DXR 30 mg/kg i.p.	32	29	25	27	28	28.2 ± 2.4 *	0.64 ± 0.10 *

Values represent the mean ± SD. * Significantly different from the negative control (*p* < 0.05); # Significantly different from the DXR (*p* < 0.05). In control, mice received saline 0.9% *per os*. MNPCE: micronucleated polychromatic erythrocytes; M: mice; DXR: Doxorubicin (positive control). PCE: polychromatic erythrocytes. NCE: normochromatic erythrocytes.

**Table 6:** Mean frequency of damaged cells, average distribution between the classes of damage and average scoring for the assessment of genotoxicity of mice treated with CEECP and respective controls.

Treatments and Cells Analyzed	Total ^1^	Comet Class				Scores
		0	1	2	3	
Peripheral blood (24 h sample) Negative control	1.78 ± 0.18 #	98.22 ± 6.65 #	1.36 ± 0.14 #	0.42 ± 0.06 #	0.00 ± 0.00 #	1.35 ± 0.14 #
CEECP 2000 mg/kg	2.02 ± 0.24 #	97.98 ± 4.74 #	1.51 ± 0.18 #	0.51 ± 0.05 #	0.00 ± 0.00 #	1.44 ± 0.10 #
DXR 30 mg/kg	77.46 ± 5.21 *	22.54 ± 1.87 *	60.57 ± 4.78 *	14.44 ± 2.33 *	2.45 ± 0.20 *	79.22 ± 6.11 *

Values represent the mean ± SD. * Significantly different from the negative control (*p* < 0.05). # Significantly different from the DXR (*p* < 0.05). In control, mice received saline 0.9% *per os*. ^1^ Total number of damaged cells (class 1 + 2 + 3). DXR: Doxorubicin (positive control).

## Data Availability

The original contributions presented in this study are included in the article/supplementary material. Further inquiries can be directed to the corresponding author.

## Supplementary Materials

The following supporting information can be downloaded at: https://www.mdpi.com/article/10.3390/plants15101508/s1, Figure S1. Base peak chromatogram of the crude ethanolic extract of the stem bark of *Cenostigma pluviosum* by HPLC–ESI–MS^n^; Figure S2. ^1^H NMR spectrum of caesalpinioflavone in CD_3_OD; Figure S3. ^13^C NMR spectrum of caesalpinioflavone in CD_3_OD; Figure S4. ^1^H-^13^C HMBC spectrum of caesalpinioflavone in CD_3_OD; Figure S5. ^1^H-^13^C HSQC spectrum of caesalpinioflavone in CD_3_OD; Table S1. ^1^H and ^13^C NMR data of Caesalpinioflavone (400 and 100MHz, CD_3_OD) compared with literature.
