# Supplementary material for: Phytochemical Profile and In Vivo Assessment of Toxicity and Anti-Inflammatory Activity of Cenostigma pluviosum var. peltophoroides (Benth.) Gagnon & G.P. Lewis
Source: Plants (Basel). 2026 May 15;15(10):1508. doi: 10.3390/plants15101508 (PMC13210627; doi:10.3390/plants15101508)
Supplement: Supplementary file 1 [file plants-15-01508-s001.zip › plants-4276102-supplementary.pdf]

*Article*

# Phytochemical Profile and In Vivo Assessment of Toxicity and Anti-Inflammatory Activity of *Cenostigma pluviosum* var. *peltophoroides* (Benth.) Gagnon & G.P. Lewis

**Natanael Teles Ramos de Lima [1], Gabriela Ribeiro de Sousa [2], Gustavo Gomes da Silva [3], Geovana Ferreira Guedes Silvestre [1], Alan Ferreira Alves [1], Ivana Maria Fechine [4], Maria de Fatima Agra [5], Alisson Macário de Oliveira [3], Josean Fechine Tavares [1,6], Marcelo Sobral da Silva [1,6] and José Maria Barbosa Filho [1,6,*]**

1

Postgraduate Program in Natural and Synthetic Bioactive Products, Federal University of Paraíba, João Pessoa 58051-900, Brazil; nteles@ltf.ufpb.br (N.T.R.d.L.); geovana.silvestre@ltf.ufpb.br (G.F.G.S.); alvesalanx@gmail.com (A.F.A.); josean@ltf.ufpb.br (J.F.T.); marcelosobral.ufpb@gmail.com (M.S.d.S.)

2

Postgraduate Program in Pharmaceutical Sciences, State University of Paraíba, Campina Grande 58429-500, Brazil; grsousafarm@gmail.com

3

Department of Pharmacy, Federal University of Rio Grande do Norte, Natal 59078-970, Brazil; gustavogmaltas@gmail.com (G.G.d.S.); alisson.macario@ufpe.br (A.M.d.O.)

4

Department of Pharmacy, State University of Paraíba, Campina Grande 58429-500, Brazil; ivana.fechine@servidor.uepb.edu.br

5

Program of Biotechnology, Center for Biotechnology, Federal University of Paraíba, João Pessoa 58051-900, Brazil; agramf@ltf.ufpb.br

6

Department of Pharmaceutical Sciences, Health Sciences Centre, Federal University of Paraíba, João Pessoa 58051-900, Brazil

*Correspondence: jbarbosa@ltf.ufpb.br

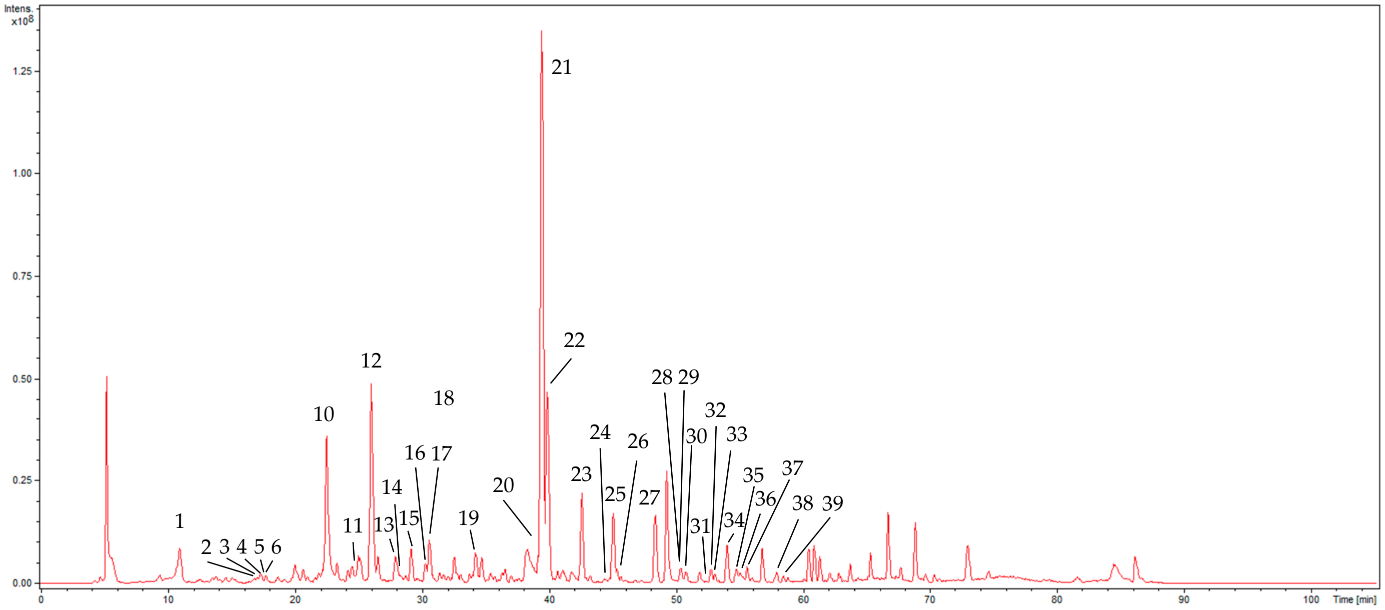


**Figure S1.** Base peak chromatogram of the crude ethanolic extract of the stem bark of *Cenostigma pluviosum* by HPLC–ESI–MS$^n$.

Isolation and structural characterization of caesalpinioflavone

Part of the crude ethanolic extract (60 g) was solubilized in a solution of H2O:MeOH 7:3 and left to stir mechanically for 30 minutes. A liquid-liquid partition was carried out using hexane, dichloromethane, ethyl acetate and n-butanol. The dichloromethane fraction was repeatedly dispersed in acetonitrile, homogenized in an ultrasonic bath, and centrifuged until a colorless supernatant was obtained, which was then concentrated under reduced pressure at 40 °C to afford 920 mg of the defatted dichloromethane fraction (DDF). The DDF was subjected to vacuum liquid chromatography (VLC) using silica gel flash as the stationary phase (SiliaFlash® F60 from SiliCycle®, 40-63 µm, 230-400 mesh, 60 Å) and pure eluents and/or binary mixtures: hexane, dichloromethane, ethyl acetate and methanol, and 11 fractions were obtained (DDF 1-11). Caesalpinioflavone was isolated using a Shimadzu preparative HPLC comprising an SLC-10A vp system controller, two LC-6AD pumps, Rheodyne manual injector and SPD-M10A DAD detector. Separation was carried out on a YMC-Actus Triart C18 (250 mm x 20.0 mm and 5 µm particles) using type l ultrapure water as solvent A and HPLC-grade acetonitrile as solvent B at a flow rate of 8 mL.min$^{-1}$. Fraction DDF-05 was purified by preparative HPLC using a gradient elution of 5–30% B (0.01–5.0 min), 30–80% B (5.0–60.0 min), 80–100% B (60.0–65.0 min), held at 100% B (65–85 min), followed by re-equilibration to 5% B (85.0–90.0 min) and maintained at 5% B (90.0–110.0 min), affording caesalpinioflavone (9.0 mg, $t_R$ = 39.7 min) (Table S1). The 1D and 2D Nuclear Magnetic Resonance spectra were obtained on Bruker Avance III Hb spectrometer at 400 MHz (Bruker Co. Ltd., Bremen, Germany). The chemical shifts (δ) are expressed in parts per million (ppm) and the coupling constants *J* in Hz, using the d (duplet), dd (double duplet) and t (triplet) to indicate the multiplicities of the $^1$H signals.

**Table S1.** $^1H$ and $^{13}C$ NMR data of Caesalpinioflavone (400 and 100MHz, $CD_3OD$) compared with literature.

| Compound 1 | | | Caesalpinioflavone [78] | | |
|---|---|---|---|---|---|
| **C** | $\delta_C$ | $\delta_H$ | **C** | $\delta_C$ | $\delta_H$ |
| **2** | 165.6 | - | **2** | 165.8 | - |
| **3** | 120.7 | - | **3** | 120.9 | - |
| **4** | 182.5 | - | **4** | 182.6 | - |
| **5** | 166.0 | - | **5** | 165.9 | - |
| **6** | 99.9 | 6.18 (d, 2.4) | **6** | 100.1 | 6.19 (d, 2.5) |
| **7** | 165.8 | - | **7** | 166.3 | - |
| **8** | 94.5 | 6,22 (d, 2.0) | **8** | 94.6 | 6.24 (d, 2.5) |
| **9** | 159.1 | - | **9** | 159.2 | - |
| **10** | 104.9 | - | **10** | 104.9 | - |
| **1′** | 124.5 | - | **1′** | 124.6 | - |
| **2′** | 116.2 | 6,81 (d, 8.4) | **2′** | 116.3 | 6.82 (d, 8.7) |
| **3′** | 131.4 | 7,01 (d, 8.8) | **3′** | 131.5 | 7.02 (d, 8.7) |
| **4′** | 160.9 | - | **4′** | 161.1 | - |
| **5′** | 131.4 | 7.01 (d, 8.8) | **5′** | 131.5 | 7.02 (d, 8.7) |
| **6′** | 116.2 | 6.81 (d, 8.4) | **6′** | 116.3 | 6.82 (d, 8.7) |
| **2′′** | 48.8[a] | 4.68 (t, 6.8) | **2′′** | 49.2 | 4.69 (dd, 7.5; 6.5) |
| **3a′′** | 35.9 | 3.44 (dd, 14.0; 6.4) | **3a′′** | 36.0 | 3.46 (dd, 14.0; 6.5) |
| **3b′′** | 35.9 | 3.05 (dd, 14.0; 7.2) | **3b′′** | 36.0 | 3.09 (dd, 14.0; 7.5) |
| **4′′** | 203.4 | - | **4′′** | 203.5 | - |
| **5′′** | 132.5 | 7.24 d (9.2) | **5′′** | 132.1 | 7.25 (d, 9.0) |
| **6′′** | 108.4 | 6.13 dd (8.8; 2.4) | **6′′** | 108.5 | 6.15 (dd, 9.0; 2.5) |
| **7′′** | 165.6 | - | **7′′** | 163.0 | - |
| **8′′** | 103.8 | 6.19 (d, 2.4) | **8′′** | 103.9 | 6.19 (d, 2.5) |
| **9′′** | 165.6 | - | **9′′** | 163.3 | - |
| **10′′** | 113.6 | - | **10′′** | 113.7 | - |
| **1′′′** | 132.0 | - | **1′′′** | 132.1 | - |
| **2′′′** | 116.0 | 6.63 (d, 8.4) | **2′′′** | 116.1 | 6.64 (d, 8.5) |
| **3′′′** | 131.3 | 6.96 (d, 8.8) | **3′′′** | 131.4 | 6.95 (d, 8.5) |
| **4′′′** | 156,7 | - | **4′′′** | 156.8 | - |
| **5′′′** | 131.3 | 6.96 (d, 8.8) | **5′′′** | 131.4 | 6.95 (d, 8.5) |
| **6′′′** | 116.0 | 6.63 (d, 8.4) | **6′′′** | 116.1 | 6.64 (d, 8.5) |

[a]Assigned by $^1H$-$^{13}C$ HSQC heteronuclear correlation spectrum.

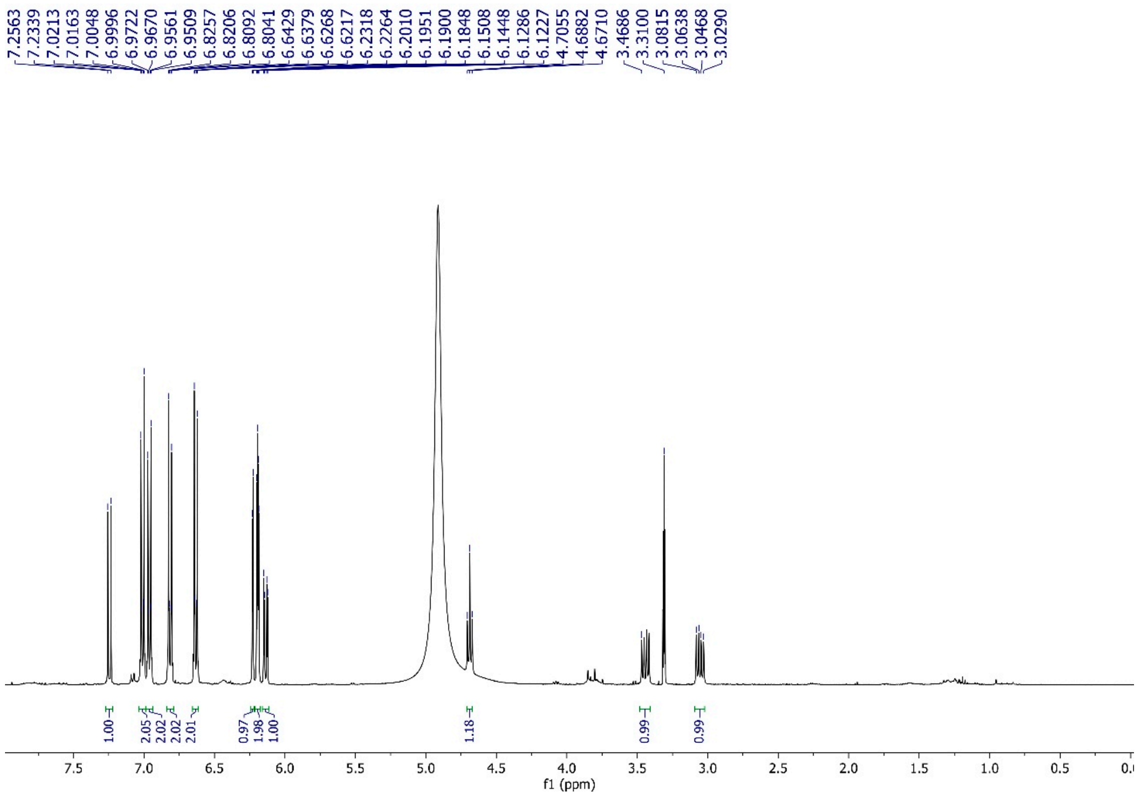


**Figure S2.** $^{1}H$ NMR spectrum of caesalpinioflavone in $CD_3OD$.

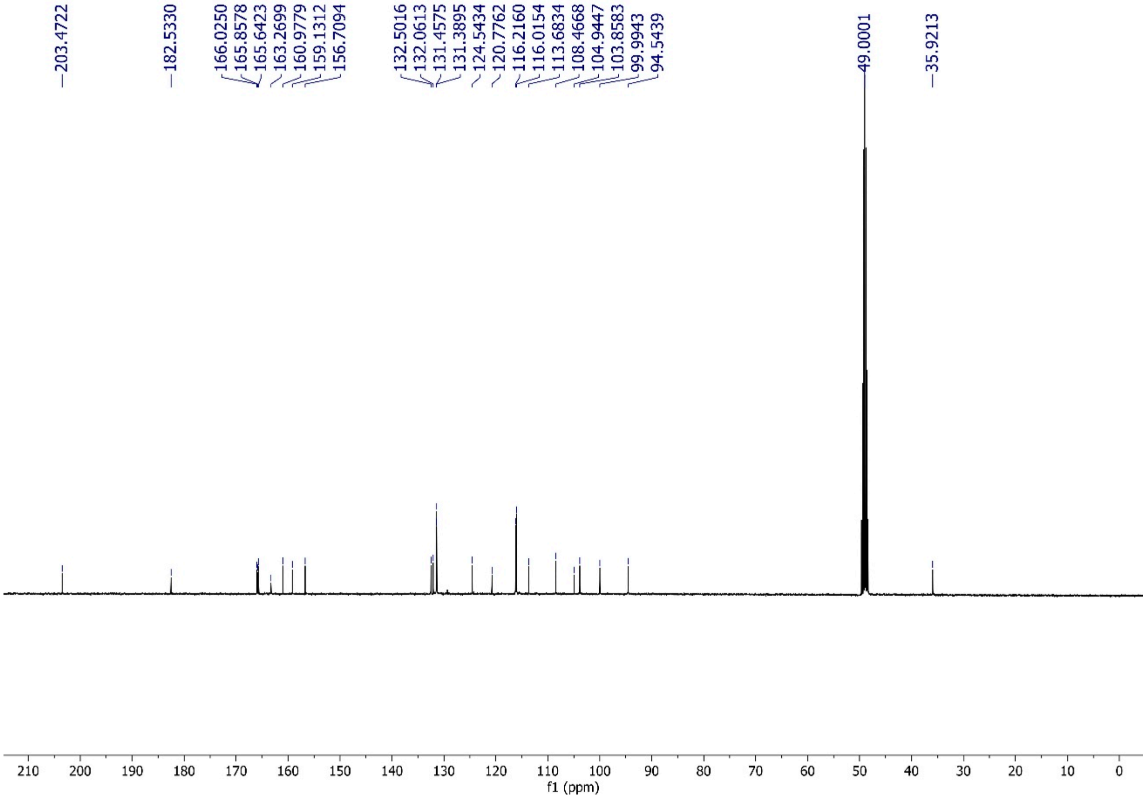


**Figure S3.** $^{13}C$ NMR spectrum of caesalpinioflavone in $CD_3OD$.

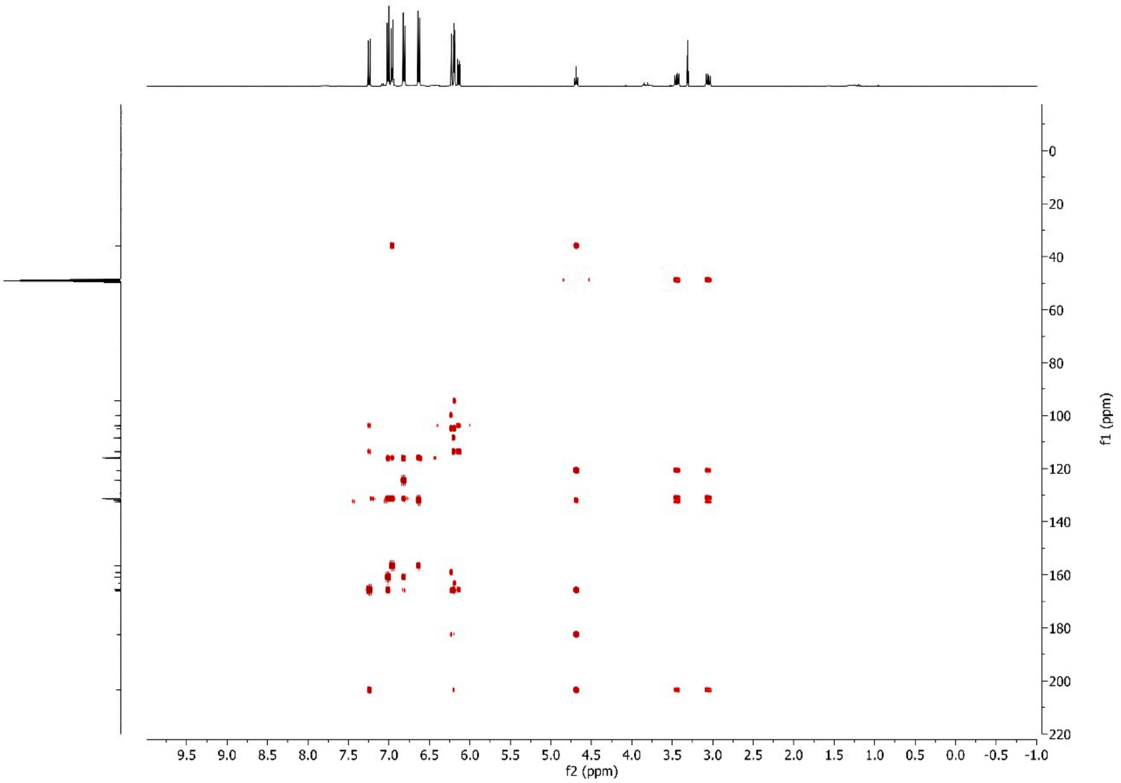


**Figure S4.** $^{1}H$-$^{13}C$ HMBC spectrum of caesalpinioflavone in $CD_3OD$.

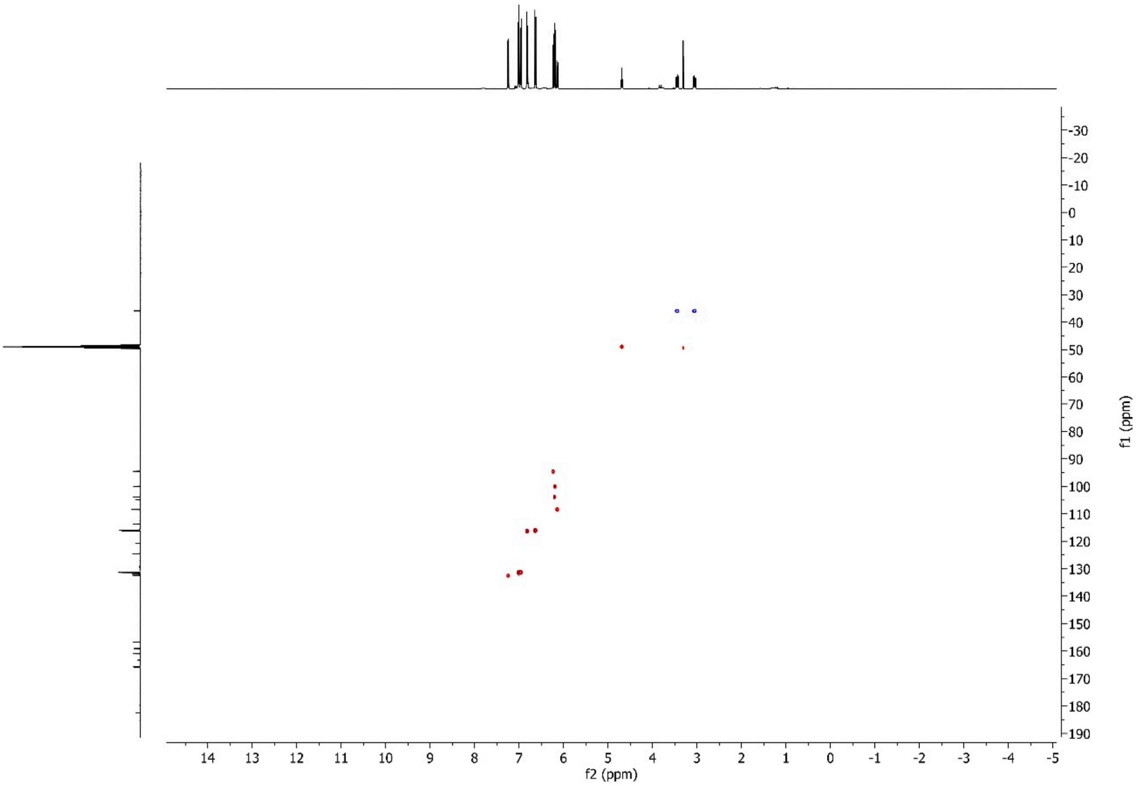


**Figure S5.** $^{1}H$-$^{13}C$ HSQC spectrum of caesalpinioflavone in $CD_3OD$.
